# Factors associated with symptom relief in patients with irritable bowel syndrome: a retrospective cohort study

**DOI:** 10.3389/fmed.2025.1643177

**Published:** 2025-08-01

**Authors:** Ming Zhao, Rui Luo, Yang Shen, Youjian Zhang, Jiaqi Dong, Muhan Lv

**Affiliations:** ^1^Department of Gastroenterology, The Affiliated Hospital, Southwest Medical University, Luzhou, China; ^2^Department of Gastroenterology, People’s Hospital of Deyang City, Deyang, China

**Keywords:** IBS, symptom relief, lifestyle factors, psychological factors, cohort study

## Abstract

**Background:**

Symptom relief in irritable bowel syndrome (IBS) varies widely, influenced by demographic, clinical, and psychosocial factors. Identifying factors associated with symptom relief can enhance management strategies for IBS patients.

**Objective:**

This study aimed to examine factors associated with symptom relief in a cohort of IBS patients, focusing on the role of demographic, lifestyle, psychological, and physiological characteristics.

**Methods:**

We conducted a retrospective cohort study of 278 IBS patients treated at a tertiary hospital. Demographic data, IBS subtype, symptom severity, lifestyle factors, and psychological variables (anxiety, depression, stress) were collected. Symptom relief, defined as a 50% reduction in baseline symptom severity, was the primary outcome. Univariate and multivariate logistic regression analyses were performed to identify factors associated with symptom relief, with subgroup analyses examining key demographic and lifestyle factors.

**Results:**

In multivariate analysis, symptom severity (OR = 1.20, 95% CI: 1.05–1.37, *p* = 0.008), daily exercise (OR = 1.50, 95% CI: 1.01–2.24, *p* = 0.04), and low depression scores (OR = 0.93, 95% CI: 0.89–0.98, *p* = 0.003) remained independent predictors of symptom relief.

**Conclusion:**

Symptom severity, daily exercise, and lower depression levels are independently associated with symptom relief in IBS patients, highlighting the importance of lifestyle modifications and mental health support. These findings suggest that physical activity and psychological well-being were associated with symptom relief, highlighting their potential relevance in holistic IBS management strategies.

## Introduction

Irritable bowel syndrome (IBS) is a prevalent functional gastrointestinal disorder marked by abdominal pain, bloating, and altered bowel habits, affecting approximately 11% of the global population (1, 2). This condition places a significant burden on patients’ quality of life and healthcare systems due to frequent consultations and the need for ongoing management (3, 4). Despite extensive research, the pathophysiology of IBS remains incompletely understood, with current evidence suggesting a multifactorial basis involving genetic predisposition, disruptions in the gut-brain axis, altered gut microbiota, and psychosocial influences (5, 6).

Symptom relief in IBS is notoriously variable, with many patients experiencing inconsistent responses to standard treatments. Studies increasingly suggest that demographic factors (such as age and gender) and lifestyle factors (such as physical activity and dietary habits) may influence symptom outcomes in IBS (7–9). Moreover, psychological comorbidities, including anxiety, depression, and stress, are commonly observed in IBS patients and are known to worsen symptom severity (10, 11). High levels of perceived stress and inadequate social support, for example, have been linked to poorer outcomes, highlighting the potential importance of addressing both physiological and psychosocial aspects of IBS.

Although several studies have explored the role of demographic and clinical predictors of symptom improvement, few have comprehensively examined the combined impact of lifestyle, psychological, and physiological factors within a single IBS cohort. Therefore, this study aims to identify factors associated with symptom relief in a well-defined cohort of IBS patients by examining a wide range of potentially modifiable predictors. By clarifying these associations, we aim to contribute to the development of more personalized, effective management strategies for IBS, ultimately improving patient outcomes and quality of life.

## Methods

### Study design and population

This retrospective cohort study was conducted among patients diagnosed with irritable bowel syndrome (IBS) at a tertiary hospital between January 2023 and July 2024. A total of 278 patients met the inclusion criteria, which required a diagnosis of IBS according to the Rome IV criteria and a minimum follow-up period of 12 months. Exclusion criteria included patients with severe psychiatric disorders, significant organic gastrointestinal diseases, or incomplete medical records.

### Data collection

Data were collected from electronic medical records and structured follow-up assessments. Variables collected included demographic information (age, gender, body mass index [BMI]), clinical characteristics (IBS subtype, symptom duration, symptom severity), lifestyle factors (smoking, alcohol use, exercise frequency), psychological variables (anxiety, depression, and stress levels), physiological measures (C-reactive protein [CRP] levels, fecal test results, gut microbiota diversity index), and medication adherence. Symptom severity was evaluated using a standardized 1–5 scale based on a global physician-administered question at the time of initial assessment, where 1 represented minimal symptoms and 5 represented very severe symptoms. This score was recorded in the electronic medical records as part of routine clinical documentation.

### Lifestyle factors

Lifestyle information included smoking status (current vs. non-smoker), alcohol consumption (regular vs. none), and exercise frequency. Exercise frequency was categorized as: none, 1–2 times/week, 3–5 times/week, and daily.

For the purposes of this study, “daily exercise” was operationally defined as engaging in at least 30 min of moderate-intensity physical activity per day, consistent with the World Health Organization (WHO) recommendations for adults. Moderate-intensity exercise includes activities such as brisk walking, cycling, or recreational swimming that elevate heart rate and respiratory effort without causing exhaustion. This classification was based on retrospective documentation in medical records and patient-reported activity levels during follow-up interviews. Although exact exercise duration and type were not always uniformly recorded for less frequent categories, patients reporting daily exercise were confirmed to meet the minimal WHO-defined criteria for aerobic activity.

### Psychological variables

Psychological assessments included anxiety, depression, and perceived stress levels. These were retrospectively extracted from structured clinical evaluations conducted at the time of diagnosis and during follow-up visits. These assessments were administered systematically to all patients as part of the hospital’s standardized evaluation pr otocol for patients with functional gastrointestinal disorders. All scores were recorded in the electronic medical system by trained clinicians and cross-referenced for accuracy. Anxiety and depression were assessed using the Hospital Anxiety and Depression Scale (HADS), a validated 14-item instrument widely used in clinical settings. Each subscale (HADS-A for anxiety and HADS-D for depression) contains 7 items scored from 0 to 3, with total subscale scores ranging from 0 to 21. Scores of 0–7 indicate normal range, 8–10 borderline abnormal, and ≥11 suggest clinically significant anxiety or depression.

Perceived stress was measured using the Perceived Stress Scale-10 (PSS-10), which includes 10 items rated on a 5-point Likert scale (0 = never to 4 = very often), yielding a total score ranging from 0 to 40. Higher scores indicate greater perceived stress, with thresholds defined as low (0–13), moderate (14–26), and high stress (27–40). Both HADS and PSS-10 have been previously validated in Chinese populations and demonstrate good internal consistency and construct validity. In this study, these scores were recorded by clinicians trained in psychological screening and were cross-referenced with electronic medical records for accuracy.

### Outcomes

The primary outcome was symptom relief, defined as a patient-reported improvement of at least 50% in overall IBS symptoms compared to baseline, sustained over the final 3 months of follow-up. Symptom changes were assessed during scheduled follow-up visits at 6 and 12 months using structured interviews conducted by trained clinicians. Patients were asked to retrospectively evaluate the change in their abdominal pain, bloating, and bowel habit abnormalities using a 5-point Likert scale ranging from “no improvement” to “complete resolution.” A response of “much improved” or “completely resolved” was considered indicative of ≥50% symptom relief. This subjective assessment was corroborated with the clinical judgment of attending gastroenterologists and was cross-referenced with symptom descriptions recorded in the electronic medical records. Although validated scoring systems such as the IBS Symptom Severity Score (IBS-SSS) were not uniformly recorded due to the retrospective design, they were not systematically implemented in clinical practice during the study period. Consequently, we relied on global self-reported symptom improvement as documented during structured follow-up interviews and corroborated by clinician judgment. We acknowledge that this subjective outcome measure may introduce recall and reporting biases, and we recommend that future prospective studies incorporate validated instruments like the IBS-SSS to improve the reliability and comparability of findings.

### Statistical analysis

Descriptive statistics were calculated for all baseline characteristics. Continuous variables are presented as mean ± standard deviation (SD), and categorical variables as frequencies and percentages. Univariate analyses were performed using logistic regression to assess the association between each variable and symptom relief, with odds ratios (ORs), 95% confidence intervals (CIs), and *p*-values reported. Variables with a p-value <0.10 in the univariate analysis were included in the multivariate logistic regression model to identify independent predictors of symptom relief, adjusting for potential confounders. The performance of the multivariate logistic regression model was evaluated using several metrics. Model discrimination was assessed using the area under the receiver operating characteristic curve (AUC). AUC values between 0.7 and 0.8 were considered acceptable, and values above 0.8 indicated good discriminative ability. Model calibration was evaluated using the Hosmer–Lemeshow goodness-of-fit test, where a *p*-value >0.05 was interpreted as a good model fit. Additionally, the Nagelkerke R^2^ statistic was calculated to assess the proportion of variance explained by the model.

Prior to conducting the multivariate logistic regression, we performed diagnostic checks for multicollinearity among the independent variables. Variance inflation factors (VIFs) were calculated for all covariates included in the multivariate model. A VIF value exceeding 5 was considered indicative of significant multicollinearity. In our analysis, all VIFs ranged from 1.05 to 2.20, indicating no evidence of problematic multicollinearity. This ensures that the estimated effects of individual predictors, such as symptom severity and depression scores, are not substantially biased by intercorrelation.

Subgroup analyses were conducted to explore whether the associations between baseline characteristics and symptom relief differed across specific strata, including gender (male vs. female), age (<45 vs. ≥45 years), IBS subtype (IBS-C, IBS-D, IBS-M), symptom severity (low vs. moderate-to-high), exercise frequency (none, 1–2/week, 3–5/week, daily), stress level (low, moderate, high), and family support (poor, average, good). These analyses aimed to identify potential effect modifiers and provide hypothesis-generating insights. All subgroup analyses were performed using SPSS (version 26.0), with statistical significance set at *p* < 0.05. Given the multiple comparisons involved, no formal adjustment (e.g., Bonferroni correction) was applied, and findings should be interpreted with caution. The subgroup analyses were exploratory in nature and not powered for definitive interaction testing.

## Results

### Baseline characteristics of IBS patients

Table 1 summarizes the baseline characteristics of the 278 IBS patients included in the study. The mean age of participants was 45.0 years (SD ± 12.0), with a higher proportion of females (60.0%) than males (40.0%). The average BMI was 24.0 kg/m^2^ (SD ± 4.0), and patients were categorized by IBS subtype: IBS-C (30.2%), IBS-D (39.9%), and IBS-M (29.9%). The mean duration of IBS symptoms was 10.5 years (SD ± 5.8), with an average symptom severity score of 3.0 (SD ± 1.2) on a scale of 1 to 5. Lifestyle factors revealed that 20.1% of patients reported smoking, and 29.9% reported alcohol use. Physical activity varied, with 24.8% engaging in no regular exercise, while 10.4% exercised daily. Psychological measures indicated moderate levels of anxiety and depression, with mean scores of 10.2 (SD ± 5.8) and 12.5 (SD ± 7.0), respectively. Stress levels were predominantly moderate (50.0%), with 20.1% reporting high stress.

**Table 1:** Baseline characteristics of IBS patients (*N* = 278).

Variable	Category	*n* (%) or Mean ± SD
Age (years)	-	45.0 ± 12.0
Gender	Male	111 (40.0%)
	Female	167 (60.0%)
BMI (kg/m^2^)	-	24.0 ± 4.0
IBS Type	IBS-C	84 (30.2%)
	IBS-D	111 (39.9%)
	IBS-M	83 (29.9%)
Symptom Duration (years)	-	10.5 ± 5.8
Symptom Severity	1–5	3.0 ± 1.2
Smoking Status	Yes	56 (20.1%)
	No	222 (79.9%)
Alcohol Use	Yes	83 (29.9%)
	No	195 (70.1%)
Exercise Frequency	None	69 (24.8%)
	1–2 times/week	97 (34.9%)
	3–5 times/week	83 (29.9%)
	Daily	29 (10.4%)
Anxiety Score	-	10.2 ± 5.8
Depression Score	-	12.5 ± 7.0
Stress Level	Low	83 (29.9%)
	Moderate	139 (50.0%)
	High	56 (20.1%)
CRP Level (mg/L)	-	5.0 ± 2.0
Fecal Test Result	Positive	42 (15.1%)
	Negative	236 (84.9%)
Gut Microbiota Diversity Index	-	2.5 ± 0.5
Medication Use	Yes	139 (50.0%)
	No	139 (50.0%)
Adherence Level	Low	56 (20.1%)
	Moderate	139 (50.0%)
	High	83 (29.9%)
Side Effects	None	167 (60.0%)
	Mild	69 (24.8%)
	Moderate	28 (10.1%)
	Severe	14 (5.1%)
Family History of IBS	Yes	84 (30.2%)
	No	194 (69.8%)
Financial Burden	Low	111 (40.0%)
	Moderate	111 (40.0%)
	High	56 (20.1%)
Family Support	Poor	28 (10.1%)
	Average	139 (50.0%)
	Good	111 (40.0%)

This table shows baseline characteristics of 278 IBS patients. Continuous variables are shown as mean ± SD; categorical variables as counts and percentages. IBS subtypes include IBS-C, IBS-D, and IBS-M. Symptom severity is rated 1–5; higher scores indicate greater severity. CRP denotes C-reactive protein levels, and the Gut Microbiota Diversity Index approximates microbial diversity.

Physiologically, patients had a mean CRP level of 5.0 mg/L (SD ± 2.0), and fecal test results were negative in 84.9% of cases. The average Gut Microbiota Diversity Index was 2.5 (SD ± 0.5), indicating a moderate range of microbial diversity. Half of the patients (50.0%) reported medication use, with varying levels of adherence: 20.1% low, 50.0% moderate, and 29.9% high. Among those on medication, 60.0% reported no side effects, while 10.1% experienced moderate effects. Family history of IBS was noted in 30.2% of patients. Financial burden was reported as low in 40.0% of cases, with moderate and high burdens in 40.0 and 20.1% of cases, respectively. Family support was generally favorable, with 50.0% rating it as average and 40.0% as good.

We further analyzed the impact of combined smoking and alcohol use on symptom severity. As shown in Supplementary Table S1, 64 out of 278 patients (23.0%) reported both smoking and regular alcohol consumption. This subgroup demonstrated a significantly higher mean symptom severity score (3.4 ± 1.0) compared to patients who engaged in neither behavior (2.8 ± 1.1, *p* = 0.01). Patients who reported only smoking (*n* = 18) or only alcohol use (*n* = 44) had intermediate severity scores (3.1 ± 1.0 and 3.0 ± 1.1, respectively), though these differences did not reach statistical significance. These findings suggest a potential additive effect of these lifestyle factors on IBS symptom burden.

### Univariate and multivariate analysis of factors associated with symptom relief in IBS patients

Table 2 presents the results of univariate and multivariate analyses examining factors associated with symptom relief in IBS patients. In the univariate analysis, several variables were significantly associated with symptom relief. Female gender (OR = 1.35, 95% CI: 1.02–1.79, *p* = 0.04) was more likely to report symptom relief compared to males. Among IBS subtypes, patients with IBS-M showed a higher likelihood of symptom relief compared to those with IBS-C (OR = 1.50, 95% CI: 1.07–2.10, *p* = 0.02). Increased symptom severity was also associated with higher odds of symptom relief (OR = 1.25, 95% CI: 1.10–1.42, *p* < 0.001). Lifestyle factors were relevant in the analysis. Daily exercise was positively associated with symptom relief compared to no exercise (OR = 1.60, 95% CI: 1.05–2.42, *p* = 0.03). Psychological factors also played a significant role; higher depression scores were inversely associated with symptom relief (OR = 0.92, 95% CI: 0.89–0.96, *p* < 0.001), and high stress levels were less likely to be associated with relief (OR = 0.60, 95% CI: 0.42–0.86, *p* = 0.005). Notably, good family support also appeared beneficial, showing a positive association with symptom relief (OR = 1.50, 95% CI: 1.01–2.23, *p* = 0.04). In the multivariate analysis, factors independently associated with symptom relief included symptom severity (OR = 1.20, 95% CI: 1.05–1.37, *p* = 0.008) and daily exercise (OR = 1.50, 95% CI: 1.01–2.24, *p* = 0.04). Higher depression scores remained negatively associated with symptom relief (OR = 0.93, 95% CI: 0.89–0.98, *p* = 0.003), indicating that depression may significantly impact symptom outcomes. Although high stress levels showed borderline significance in the multivariate model (OR = 0.70, 95% CI: 0.49–1.00, *p* = 0.05), good family support maintained a positive association with symptom relief (OR = 1.40, 95% CI: 1.00–2.10, *p* = 0.05). The multivariate logistic regression model demonstrated acceptable discriminative ability, with an AUC of 0.76 (95% CI: 0.71–0.80). The Hosmer–Lemeshow test yielded a *p*-value of 0.38, indicating a good model fit. The Nagelkerke R^2^ value was 0.32, suggesting that approximately 32% of the variability in symptom relief was explained by the included predictors. These findings support the overall robustness and predictive utility of the model. These findings suggest that symptom severity, daily physical activity, depression levels, stress, and family support are important factors in predicting symptom relief among IBS patients. Adjusting for confounding factors in the multivariate analysis highlights the significance of psychological and lifestyle factors, particularly daily exercise and family support, as potentially modifiable influences on symptom outcomes in this population.

**Table 2:** Univariate and multivariate analysis of factors associated with symptom relief in IBS patients.

Variable	Univariate analysis (OR, 95% CI, *p*-value)	Multivariate analysis (OR, 95% CI, p-value)
Age (years)	1.02 (1.01–1.03, *p* = 0.01)	1.01 (0.99–1.02, *p* = 0.12)
Gender		
Male	Reference	Reference
Female	1.35 (1.02–1.79, *p* = 0.04)	1.10 (0.78–1.56, *p* = 0.31)
BMI (kg/m^2^)	0.97 (0.92–1.02, *p* = 0.24)	-
IBS type		
IBS-C	Reference	Reference
IBS-D	1.20 (0.87–1.66, *p* = 0.26)	1.15 (0.83–1.62, *p* = 0.34)
IBS-M	1.50 (1.07–2.10, *p* = 0.02)	1.30 (0.94–1.81, *p* = 0.10)
Symptom duration (years)	1.01 (0.99–1.03, *p* = 0.14)	-
Symptom severity	1.25 (1.10–1.42, *p* < 0.001)	1.20 (1.05–1.37, p = 0.008)
Smoking status		
No	Reference	Reference
Yes	0.85 (0.60–1.20, *p* = 0.36)	-
Alcohol use		
No	Reference	Reference
Yes	1.30 (0.95–1.78, *p* = 0.09)	1.15 (0.81–1.65, *p* = 0.41)
Exercise frequency		
None	Reference	Reference
1-2 times/week	1.20 (0.85–1.68, *p* = 0.28)	1.10 (0.78–1.55, *p* = 0.48)
3-5 times/week	1.35 (0.96–1.90, *p* = 0.08)	1.20 (0.86–1.69, *p* = 0.29)
Daily	1.60 (1.05–2.42, *p* = 0.03)	1.50 (1.01–2.24, p = 0.04)
Anxiety score	0.95 (0.91–0.98, *p* = 0.004)	0.96 (0.92–1.00, *p* = 0.06)
Depression score	0.92 (0.89–0.96, *p* < 0.001)	0.93 (0.89–0.98, *p* = 0.003)
Stress level		
Low	Reference	Reference
Moderate	0.85 (0.60–1.20, *p* = 0.36)	-
High	0.60 (0.42–0.86, *p* = 0.005)	0.70 (0.49–1.00, *p* = 0.05)
CRP level (mg/L)	1.02 (0.98–1.06, *p* = 0.29)	-
Medication Use		
No	Reference	Reference
Yes	1.25 (0.92–1.70, *p* = 0.15)	-
Adherence level		
Low	Reference	Reference
Moderate	1.10 (0.78–1.55, *p* = 0.59)	-
High	1.35 (0.92–1.97, *p* = 0.12)	1.20 (0.83–1.74, *p* = 0.33)
Family support		
Poor	Reference	Reference
Average	1.20 (0.78–1.85, *p* = 0.40)	-
Good	1.50 (1.01–2.23, *p* = 0.04)	1.40 (1.00–2.10, *p* = 0.05)

The univariate analysis includes odds ratios (ORs) with 95% confidence intervals (CIs) and p-values for each variable. Significant variables (*p* < 0.05) were then included in the multivariate analysis to determine independent associations with symptom relief in IBS patients.

### Subgroup analysis of factors associated with symptom relief in IBS patients

Table 3 presents the results of the subgroup analysis, highlighting factors associated with symptom relief across various demographic, clinical, and psychosocial characteristics in IBS patients. In terms of gender, females had a slightly higher rate of symptom relief (54.5%) compared to males (45.9%), with an odds ratio (OR) of 1.35 (95% CI: 1.00–1.82, *p* = 0.05), suggesting a marginally significant association. Age did not show a significant difference, with patients younger than 45 years having a symptom relief rate of 52.5%, compared to 47.5% in those aged 45 years and above (OR = 0.90, 95% CI: 0.68–1.20, *p* = 0.47). Regarding IBS subtypes, patients with the IBS-M subtype had the highest rate of symptom relief (55.0%) compared to those with IBS-C (40.5%), with a significant OR of 1.60 (95% CI: 1.02–2.51, *p* = 0.04). Symptom severity was also significant, with patients experiencing moderate to high severity (score 3–5) showing greater symptom relief (57.7%) compared to those with low severity (42.3%) (OR = 1.50, 95% CI: 1.11–2.04, *p* = 0.01). Exercise frequency demonstrated a strong association with symptom relief. Patients who exercised daily had the highest relief rate at 65.5% (OR = 2.40, 95% CI: 1.20–4.80, *p* = 0.01), while those who exercised 3–5 times per week also showed a significant benefit (OR = 1.65, 95% CI: 1.01–2.68, *p* = 0.04) compared to those with no regular exercise. Psychosocial factors were impactful in this analysis. Patients with low stress levels had the highest rate of symptom relief (58.0%), while those with high stress levels showed significantly lower relief rates (39.0%, OR = 0.55, 95% CI: 0.31–0.96, *p* = 0.03). Family support was another notable factor; patients reporting good family support had a symptom relief rate of 60.5%, with an OR of 2.30 (95% CI: 1.14–4.62, *p* = 0.02) compared to those with poor support. Overall, these findings suggest that specific subgroups, such as those with higher exercise frequency, moderate to high symptom severity, low stress levels, and strong family support, are more likely to experience symptom relief. These insights emphasize the importance of physical activity, stress management, and social support as potential areas for targeted interventions to improve outcomes in IBS patients.

**Table 3:** Subgroup analysis of factors associated with symptom relief in IBS patients.

Subgroup	*N*	Symptom relief (%)	OR (95% CI)	*p*-value
Gender				
Male	111	45.9	Reference	-
Female	167	54.5	1.35 (1.00–1.82)	0.05
Age				
<45 years	139	52.5	Reference	-
≥45 years	139	47.5	0.90 (0.68–1.20)	0.47
IBS type				
IBS-C	84	40.5	Reference	-
IBS-D	111	50.5	1.40 (0.92–2.12)	0.12
IBS-M	83	55.0	1.60 (1.02–2.51)	0.04
Symptom severity				
Low (1–2)	111	42.3	Reference	-
Moderate to High (3–5)	167	57.7	1.50 (1.11–2.04)	0.01
Exercise frequency				
None	69	35.0	Reference	-
1-2 times/week	97	45.5	1.30 (0.82–2.06)	0.26
3-5 times/week	83	53.0	1.65 (1.01–2.68)	0.04
Daily	29	65.5	2.40 (1.20–4.80)	0.01
Stress level				
Low	83	58.0	Reference	-
Moderate	139	51.0	0.85 (0.55–1.31)	0.46
High	56	39.0	0.55 (0.31–0.96)	0.03
Family support				
Poor	28	35.0	Reference	-
Average	139	50.0	1.65 (0.82–3.32)	0.17
Good	111	60.5	2.30 (1.14–4.62)	0.02

This table presents a subgroup analysis of symptom relief by demographic, clinical, and psychosocial characteristics among IBS patients. Odds ratios (OR) with 95% confidence intervals (CIs) and *p*-values are shown for each subgroup comparison. Significant associations (*p* < 0.05) indicate notable subgroup differences in symptom relief rates, suggesting that daily exercise, high symptom severity, female gender, IBS-M subtype, low stress, and good family support are positively associated with symptom relief.

## Discussion

In this retrospective cohort study, we identified key factors associated with symptom relief in patients with irritable bowel syndrome (IBS), specifically highlighting the roles of symptom severity, physical activity, depression levels, stress, and family support. These findings provide valuable insights into factors associated with symptom relief and underscore the importance of considering holistic approaches in IBS management, while acknowledging that causal relationships cannot be established due to the study design.

Our results confirm the complex interplay between lifestyle, psychological, and physiological factors in influencing IBS outcomes. The association between higher baseline symptom severity and increased likelihood of symptom relief may initially seem counterintuitive. However, this finding aligns with prior studies suggesting that individuals experiencing more pronounced symptoms are often more motivated to adhere to therapeutic interventions and lifestyle modifications, potentially enhancing their response to treatment (12–14). Therefore, a comprehensive initial assessment of symptom severity could inform personalized care strategies, enabling targeted resource allocation and potentially improving long-term outcomes. Alternatively, the observed association between higher baseline symptom severity and greater symptom relief may partially reflect regression to the mean, a statistical phenomenon whereby individuals with extreme baseline values tend to show improvement over time, independent of actual treatment effects. This possibility should be considered when interpreting our findings and highlights the importance of incorporating control groups or repeated baseline measures in future prospective studies.

In addition to exercise, lifestyle behaviors such as smoking and alcohol consumption were found to be associated with differences in IBS symptom severity. Our analysis revealed that approximately 13.7% of patients engaged in both behaviors, and this subgroup demonstrated significantly higher symptom severity scores. Previous studies have shown that smoking and alcohol can independently and synergistically disrupt gastrointestinal motility, alter visceral sensitivity, and induce low-grade mucosal inflammation. These mechanisms may explain the observed aggravation of IBS symptoms in individuals with dual exposure. Although our study was not primarily designed to evaluate lifestyle interactions, this finding underscores the importance of targeted counseling on modifiable behaviors in IBS management. Future studies should examine the dose–response relationship and potential reversibility of symptoms following lifestyle modification in this population.

Physical activity, particularly daily exercise, was significantly associated with symptom relief. This finding aligns with previous reports that suggest exercise may play a beneficial role in IBS symptom management, although causality cannot be determined from our retrospective data (15, 16). Physical activity is hypothesized to influence gut motility, immune function, and systemic inflammation, which may in turn be associated with symptom patterns observed in IBS patients (17). While our study did not directly measure these physiological effects, the strong association between daily exercise and symptom relief suggests that regular physical activity may be considered a potentially beneficial component of IBS care, given its observed association with symptom relief.

In addition to physical activity, other lifestyle factors—particularly smoking and alcohol use—may also influence IBS symptom expression. Our analysis revealed that patients who engaged in both smoking and alcohol consumption reported the highest symptom severity scores. This aligns with previous evidence suggesting that nicotine and ethanol may synergistically disrupt intestinal motility, promote mucosal inflammation, and exacerbate visceral hypersensitivity. These agents may impair gut barrier function, alter gut microbiota composition, and activate pro-inflammatory signaling, all of which have been implicated in IBS pathogenesis. While this study was not designed to establish causality, the observed association highlights the need to include lifestyle behavior assessment as part of comprehensive IBS management. Future research should further investigate dose–response relationships and the reversibility of symptoms following cessation or reduction of these exposures.

Psychological factors, particularly depression and stress levels, were also significantly associated with symptom relief. Lower depression scores independently predicted better symptom outcomes, emphasizing the need to address mental health in IBS management. Depression and stress are known to exacerbate gastrointestinal symptoms via the gut-brain axis, suggesting that interventions targeting mental health may indirectly facilitate symptom relief (18). While psychological comorbidities are well-documented in IBS populations, our study reinforces their impact on treatment outcomes. Thus, integrating psychological support, such as cognitive-behavioral therapy (CBT) or mindfulness-based stress reduction, may offer a pathway to improved symptom control for patients with high psychological distress. The psychological assessments in this study were based on well-validated instruments (HADS and PSS-10), which have demonstrated good psychometric properties in Chinese populations, thereby supporting the reliability of our findings.

An additional noteworthy finding was the positive impact of family support on symptom relief. Good family support has previously been linked to better outcomes across various chronic conditions and appears to play a role in IBS management as well (19). Social support can improve adherence to treatment, alleviate psychological distress, and promote a sense of well-being, which may collectively contribute to better symptom control (20). Therefore, engaging family members in patient education and care planning could be a valuable component of a multifaceted treatment strategy for IBS. Despite its apparent significance, the specific mechanisms by which family support facilitates symptom relief remain unclear. Social support may buffer psychological distress and enhance behavioral adherence via the brain–gut axis. However, in this study, family support was assessed using a single categorical variable, without differentiating types of support (e.g., emotional, instrumental). Future studies should incorporate validated multidimensional social support scales to better understand its contribution to IBS outcomes. Multicollinearity diagnostics confirmed that the associations identified in the multivariate model, including those between symptom severity and depression, were not substantially influenced by inter-variable correlation, supporting the robustness of the model findings. In addition, the model demonstrated acceptable discrimination and good calibration, further supporting the reliability of the identified predictors.

This study has several notable strengths. It provides a comprehensive and integrative assessment of demographic, lifestyle, psychological, and physiological variables within a single IBS cohort, offering a more holistic understanding of factors associated with symptom relief. The inclusion of gut microbiota diversity indices and inflammatory biomarkers such as CRP adds biological depth to the predominantly clinical and psychosocial data, thereby enhancing the translational value of the findings. However, the present study did not investigate the specific microbial taxa or inflammatory pathways involved. Understanding how alterations in microbiota composition or low-grade inflammation contribute to IBS symptomatology requires further mechanistic studies. Future research should integrate microbial sequencing, metabolomics, and cytokine profiling to uncover potential biological mediators of symptom relief. In addition, mechanistic investigations should aim to elucidate how modifiable factors—such as physical activity, stress reduction, and social support—mediate symptom improvement, potentially through pathways involving modulation of systemic inflammation, alterations in gut microbiota composition, or enhancement of mood via the gut-brain axis. Moreover, the use of multivariate and subgroup analyses allowed for the identification of independent predictors while accounting for potential confounders.

However, several limitations must also be acknowledged. First, the retrospective design restricts causal inference, as temporal relationships between exposures and outcomes cannot be definitively established. For example, individuals with symptom relief may be more likely to engage in physical activity, rather than exercise being the cause of symptom improvement. Therefore, the observed associations should be interpreted with caution, and future longitudinal or interventional studies are needed to explore potential causal mechanisms. Although statistical associations were observed between lifestyle, psychological, and physiological factors and symptom relief, the temporal sequence remains ambiguous. For instance, it is unclear whether improvements in depression or increased exercise frequency occurred before or after symptom relief. To address this limitation, future prospective cohort studies or randomized controlled trials are necessary to establish temporal directionality and determine causality.

Second, the primary outcome of symptom relief was measured through a non-validated, patient-reported assessment of ≥50% improvement in symptoms. While this approach enabled retrospective outcome capture across all patients, it introduces a substantial risk of recall and reporting bias. Furthermore, the use of this subjective measure limits the comparability of our findings with studies employing standardized and validated symptom scales, such as the Irritable Bowel Syndrome Symptom Severity Score (IBS-SSS). Although we attempted to mitigate this limitation by incorporating physician interviews and structured follow-up documentation, future studies should prioritize the use of validated instruments like the IBS-SSS to improve reliability, reproducibility, and cross-study comparability. Although we attempted to standardize outcome assessment through structured interviews and physician verification, the use of subjective self-report without validated instruments such as the IBS-SSS may reduce the precision and reproducibility of the primary outcome. To mitigate potential recall bias, follow-up interviews were conducted by trained clinicians using structured questionnaires at two separate time points, and patient-reported symptom changes were cross-validated against clinical documentation in the electronic medical record. While the absence of a formally validated scoring tool like IBS-SSS is a limitation, our multi-source approach aimed to improve the consistency and reliability of outcome data. Future studies should prioritize the use of standardized symptom scoring systems to enhance the objectivity and comparability of IBS treatment outcomes. Specifically, prospective studies should incorporate validated symptom assessment instruments such as the Irritable Bowel Syndrome Symptom Severity Score (IBS-SSS) to enhance measurement consistency and cross-study reliability.

Third, data on lifestyle and psychological variables were partially extracted from clinical records and may be prone to information bias due to underreporting or inconsistencies in documentation. To reduce this bias, we verified psychological variables such as anxiety, depression, and stress using scores derived from HADS and PSS-10, which were recorded during formal clinical evaluations. Lifestyle variables, including exercise and alcohol use, were additionally confirmed through structured follow-up interviews. Nevertheless, prospective real-time data collection would improve accuracy and minimize recall and recording bias in future studies.

Fourth, selection bias may exist, as the study population was derived exclusively from a single tertiary hospital in China, which may not adequately represent patients managed in primary care settings—where the majority of IBS cases are typically encountered—or those from different cultural, geographic, or healthcare contexts. Although the hospital serves a broad regional population, the patient characteristics and clinical practices in this specialized center may differ from those in community-based or international settings. Therefore, the external validity of our findings is limited, and caution should be exercised when extrapolating these results beyond similar tertiary care environments in China. Multi-center studies involving diverse populations across urban and rural regions are recommended to enhance external validity.

Finally, although efforts were made to minimize missing data, certain variables—particularly in subgroup analyses—may have been affected by incomplete information. To address these limitations, future prospective cohort studies or interventional trials should incorporate standardized symptom assessment tools, validated psychological instruments (e.g., PHQ-9, GAD-7), and objective measures of exercise and stress. Multi-center studies with broader sampling strategies and longer follow-up periods are also needed to improve the external validity and reproducibility of these findings. In addition, integrating microbiome sequencing and biomarker profiling over time may further elucidate the dynamic interplay between physiological and psychosocial factors in IBS symptom resolution.

It is important to note that the subgroup analyses conducted in this study involved multiple comparisons, and no formal correction for multiple testing (e.g., Bonferroni adjustment) was applied. Therefore, these results should be interpreted with caution, as they may be subject to an increased risk of type I error. These analyses were exploratory in nature and intended primarily for hypothesis generation. Overall, the subgroup findings suggest that specific patient groups—such as those with higher exercise frequency, moderate to high symptom severity, low stress levels, and strong family support—are more likely to report symptom relief. Therefore, the findings from subgroup analyses should be interpreted with caution and primarily serve as hypothesis-generating rather than confirmatory evidence.

Additionally, treatments and lifestyle interventions were not standardized across participants. While we recorded medication use, exercise frequency, and psychological scores, specific treatment regimens, dietary changes, and other behavioral interventions were heterogeneous and not uniformly tracked. This variation limits our ability to attribute observed improvements to specific interventions. Future studies should adopt protocolized or stratified treatment approaches to better clarify the individual contributions of different management strategies.

In conclusion, this study identifies symptom severity, daily physical activity, lower depression scores, reduced stress, and strong family support as important factors associated with symptom relief in IBS. These findings support a multidisciplinary approach to IBS management that addresses both lifestyle and psychological needs. Clinicians should consider promoting regular exercise, providing mental health support, and involving family in the care process as part of comprehensive IBS management plans. Future research should continue exploring personalized interventions to further optimize outcomes for IBS patients, while ensuring broader inclusion of diverse healthcare settings and populations to improve generalizability. Moreover, these findings may inform the development of a stepped-care model for IBS management, in which low-cost and low-risk interventions—such as regular physical activity, psychological support, and enhanced family engagement—are implemented as initial steps before escalating to more intensive or pharmacologic treatments. Such a model could improve cost-effectiveness, minimize side effects, and promote patient-centered care.

## Data Availability

The raw data supporting the conclusions of this article will be made available by the authors, without undue reservation.
